# First *de novo* draft genome sequence of *Oryza coarctata*, the only halophytic species in the genus Oryza

**DOI:** 10.12688/f1000research.12414.2

**Published:** 2017-12-15

**Authors:** Tapan Kumar Mondal, Hukam Chand Rawal, Kishor Gaikwad, Tilak Raj Sharma, Nagendra Kumar Singh

**Affiliations:** 1 National Research Centre on Plant Biotechnology (ICAR), PUSA, New Delhi, 110012, India

**Keywords:** Abiotic stress, Genome assembly, Halophyte, Nanopore, NGS, Salt stress, Wild Oryza, Whole genome sequencing

## Abstract

*Oryza coarctata* plant, collected from Sundarban delta of West Bengal, India,  has been used in the present study to generate draft genome sequences, employing the hybrid genome assembly with Illumina reads and third generation Oxford Nanopore sequencing technology. We report for the first time the draft genome with the coverage of 85.71 %  and  deposited the raw data in NCBI SRA, with BioProject ID PRJNA396417.

## Introduction

Soil salinity is a major abiotic stress of rice cultivation globally ( Molla *et al.*, 2015), and rice cultivation areas under soil salinity stress are increasing gradually. Genetic potential for salt tolerance of rice that exists among the natural population has been largely exploited, and alternative useful alleles may further enhance salinity tolerance. Wild species are a potential source of many useful genes and QTLs that may not be present in the primary gene pool of the domesticated species.

*Oryza coarctata*, known as Asian wild rice, grows naturally in the coastal region of South-East Asian countries. It flowers and set seeds under as high as 40 E.Ce dS m ^-1^ saline soil ( Bal & Dutt, 1986). It is the only species in the genus *Oryza* that is halophyte in nature. However, with the exception of one transcriptomic ( Garg *et al.*, 2014) and one miRNA ( Mondal *et al.*, 2015) experiment, no large scale generation of any other genomic resource is available for this important species, although several pinitol biosynthesis pathway genes have been cloned to study the functional genomics ( Sengupta & Majumder, 2009).

## Methods

The plant was collected from its native place, Sundarban delta of West Bengal, India (21°.36'N and 88°.15' E) and established at our institute Net house through clonal propagation. To determine the genome size, 20 mg of young leaf tissue from Net house grown plants was chopped into small pieces and stained with RNase containing propidium iodide (50 μg/ml) (BD Science, India) as per the protocol of Dolezel *et al.* (2007). The samples were filtered through a 40-μM mesh sieve (Corning, USA), before analysis in (CFM) BD FACS Calibur (BD Biosciences, San Jose, CA, USA). *Pisium sativum* leaf was used as standard for calculating the genome size. Further, high-quality genomic DNA from 100 mg young leaf of a single plant was extracted using CTAB method ( Ganie *et al.*, 2016) for the preparation of various genomic DNA libraries. We used standard Illumina HiSeq 4000 platform (San Diego, CA, USA) to construct 151-bp paired-end libraries and four mate-pair libraries of four different sizes (average of 2, 4, 6 and 8 kb size). In addition, we also used third generation sequencing (Oxford Nanopore) technology for better assembly. Sequencing was performed on MinION Mk1b (Oxford Nanopore Technologies, Oxford, UK) using SpotON flow cell (R9.4) in a 48h sequencing protocol on MinKNOW 1.4.32. Base calling was performed using Albacore. Base called reads were processed using poRe version 0.24 ( Watson *et al.*, 2015) and poretools version 0.6.0 ( Loman & Quinlan, 2014). Assembly of the high quality reads was performed using PLATANUS v1.2.4 ( Kajitani *et al.*, 2014) and SSPACE v3.0 ( Boetzer *et al.*, 2011) with default parameter. The simple sequence repeats (SSRs) of each scaffold were identified by MISA perl script ( Thiel *et al.*, 2003). Gene model prediction was done by ab initio gene predictor AUGUSTUS 3.1 ( Stanke & Waak, 2003) and sequence evidence based annotation pipeline, MAKER v2.31.8 ( Campbell *et al.*, 2014) with *O. sativa ssp. japonica* as reference gene model. The protein-coding genes were annotated by using BLAST based approach against a database containing functional plant genes downloaded from NCBI with Blast2GO (version 4.01) ( Conesa & Gotz, 2008). Genes with significant hits were assigned with GO (Gene Ontology) terms and EC (Enzyme Commission) numbers. InterProScan search and pathway analyses with KEGG database were also performed by using Blast2GO. Non-coding RNAs, such as miRNA, tRNA, rRNA, snoRNA, snRNA, were identified by adopting Infernal v1.1.2 ( Nawrocki & Eddy, 2013) using Rfam database (release 9.1) ( Nawrocki *et al.*, 2015) and snoscan distribution. Transfer RNA was predicted using tRNAscan-SE v 1.23 ( Lowe & Eddy, 1997)

## Discussion

The *O. coarctata* genome (2n=4X=48; KKLL; Sanchez *et al.*, 2013) is self-pollinated, ( Sarkar *et al.,* 1993) tetraploid plant with a genome size estimated by flow cytometry is found to be approximately 665Mb. The Illumina 4000 GA IIx sequencer pair-end generated 123.78 Gb data. Further four mate-pair libraries together generated 36.54 Gb and Nanopore generated 6.35 Gb sequence data. Hence, we achieved 250.66 X depth of the genome of *O. coarctata*. The final assembly generated 58362 numbers of scaffolds with a minimum length of 200 bp to maximum length of 7,855,609 bp and 1,858,627 bp N50 value, making a total scaffold length of 569994164 (around 570 Mb) assembled genome, resulting in 85.71% genome coverage. It has been calculated that data contain very small amount of non-ATGC character. Further, we also found that the 19.89% of the assembled genome is repetitive in nature. We also identified approximately 5512 different non-coding RNAs and around 230,968 SSRs. Gene ontology analysis identified several salt responsive genes.

## Data availability

Raw sequence data are available at NCBI SRA under the BioProject ID: PRJNA396417.
