# Supplementary material for: First de novo draft genome sequence of Oryza coarctata, the only halophytic species in the genus Oryza
Source: F1000Res. 2017 Dec 15;6:1750. Originally published 2017 Sep 25. [Version 2] doi: 10.12688/f1000research.12414.2 (PMC5657017; doi:10.12688/f1000research.12414.2)
Supplement: Peer review history

# Peer review: Referee response for version 2

**DOI:** 10.5256/f1000research.14545.r29080

**Published:** 2017-12-27

**Authors:** Stephen P. Moose

**Affiliations:** 1 Department of Crop Sciences, University of Illinois at Urbana-Champaign, Champaign, IL, USA

The authors have included the requested details about the source of plant materials, estimate of genome size, and genome assembly methods. Although still not sure how they arrived at the estimate of 19.86% repeat sequences, this is a minor point.

I have read this submission. I believe that I have an appropriate level of expertise to confirm that it is of an acceptable scientific standard.

# Peer review: Referee response for version 1

**DOI:** 10.5256/f1000research.13443.r26359

**Published:** 2017-10-20

**Authors:** Kashmir Singh

**Affiliations:** 1 Department of Biotechnology, Panjab University, Chandigarh, India

The work describe the whole genome sequence of wild species of *Oryza coarctata* species that exclusively grow under saline water and thus will be an important source of salinity tolerance genes. These genes can later be used to introduce salinity tolerance in commercial cultivars of rice. The authors used Illumina and Oxford nanopore sequencing platforms to generate 372.48X data.

 The genome sequencing methods seems good enough but authors have discussed very little about the annotation of the genome data. I can understand that there is word limit under Data Note in F1000Research, but still by looking at the discussion, I think analysis portion is weak point in this paper. Authors should provide a comparative note on the genome of *Oryza sativa* and *Oryza coarctata. *How this species is tolerating such a high saline conditions, which kind of genes/osmoregulators are involved in this adaptation should be discussed along with comparison to *O. sativa*. How many different genes were predicted should be mentioned. Authors found approximately 1605 non-coding RNAs? I am not sure, what are trying to tell here, this number should be high as per my opinion.

 There are some minor mistakes like; in the affiliation the word “Delhi” is not required. The word, “Primary” should be  inserted in the first paragraph last line of Introduction. So the correct sentence will be "...in the primary gene pool...".

I have read this submission. I believe that I have an appropriate level of expertise to confirm that it is of an acceptable scientific standard.

# Peer review: Referee response for version 1

**DOI:** 10.5256/f1000research.13443.r26486

**Published:** 2017-10-13

**Authors:** Stephen P. Moose

**Affiliations:** 1 Department of Crop Sciences, University of Illinois at Urbana-Champaign, Champaign, IL, USA

The authors report a whole genome sequence dataset for a halophytic wild rice species. These data will be useful for discovery of novel alleles for rice improvement, and for comparative/evolutionary genomics within the Oryza genus. 

1. The report would benefit from more details on the plant accession used as source of DNA for sequencing. It is stated O. coarctata is tetraploid. Was that determined by the authors, or is there a citation to include? Is it known whether O. coarctata is typically self or cross-pollinated, or other information about expected degree of heterozygosity? When grown in greenhouse to generate the plant tissue used for DNA extraction, were the plant(s) established from seeds, or via clonal propagation? Was the genomic DNA used to prepare sequencing libraries from a single plant, or a pool from multiple plants? This information is important to assess expected frequencies of variant types such as alleles or homeologs due to tetraploidy, which are likely collapsed to varying degrees in the subsequent assembly.
2. There is mention of an assembly and its quality, but not about the method(s) used to produce it or key parameters that guided the assembly. Can the authors provide that information, so that others have a benchmark upon which to compare future assemblies using the datasets?
3. The sentence “Further, we also found that the repeat contain 19.89% of the genome.” Is not completely clear. I believe what the authors intend to say is that approximately 20% of the genome assembly is comprised of repeats. How was this sequence fraction defined as repeats, via tool for matching to known repeat sequences, or a de novo approach? By inference, it is also likely that the approximately 100-kb of the estimated genome size not covered by the assembly is comprised of high-copy repeats, leading to an estimate of about 30% total repeat content.

I have read this submission. I believe that I have an appropriate level of expertise to confirm that it is of an acceptable scientific standard, however I have significant reservations, as outlined above.

# Peer review: Referee response for version 1

**DOI:** 10.5256/f1000research.13443.r26360

**Published:** 2017-10-11

**Authors:** Sandip Das

**Affiliations:** 1 Department of Botany, University of Delhi, Delhi, India

The authors report a draft genome sequence of a halophyte Oryza species collected from Sunderbans, and provides a glimpse into the adaptive strategies employed by Oryza against salinity stress. Undoubtedly, it will be an useful resource for future functional characterization, comparative genomic studies, and developing salinity tolerance in rice. I understand that the present format is only for reporting, and look forward to reading the full manuscript with all the analysis. There are small language edits that that authors need to incorporate.

 Comments:

 Please add a reference or sufficient information for general readers as to how genome types of rice (for instance, KKLL in case of *O. coarctata*) was assigned.

 Language corrections:

1. Change “have been used” to “has been used”
2. Change “We report for the first time that more than 85.71 % of the genome coverage and the data have been deposited in NCBI SRA, with BioProject ID PRJNA396417” to “deposited in NCBI SRA, with BioProject ID PRJNA396417”
3. Change “and established to our institute NET” to “and established at our institute NET”
4. Change “resulting 85.71 % genome coverage” to “resulting in 85.71 % genome coverage”
5. Change “we also found that the repeat contain 19.89% of the genome.” to “we also found that the 19.89% of the genome is repetitive in nature”.

I have read this submission. I believe that I have an appropriate level of expertise to confirm that it is of an acceptable scientific standard.
